# Audit of strategies to improve sepsis management in emergency departments

**DOI:** 10.1186/cc14085

**Published:** 2015-03-16

**Authors:** CE Thakker, P Crook, B Davies, L Mawer, T Tomouk, E Williams, C Gray, K Turner

**Affiliations:** 1 University of Cambridge, UK; 2 Ipswich Hospital, Ipswich, UK

## Introduction

Severe sepsis results in ~36,800 UK deaths each year [1]. Prior studies demonstrate the benefit of early recognition and treatment of sepsis in reducing mortality [2]. The Sepsis Six [1] bundle aims to optimise the first hour of sepsis management. We assessed the proportion of emergency department (ED) patients with severe sepsis receiving the Sepsis Six bundle and whether this was improved by a combination of staff education and use of Sepsis Six management stickers in patient notes.

## Methods

A closed loop audit was completed in the ED at Ipswich Hospital, UK. Each cycle was 14 days with interventions made in a 4-week period between the two cycles. The interventions consisted of: Sepsis Six management stickers and posters placed in the ED; two training sessions for all ED nurses on sepsis recognition and management; a teaching session for all middle-grade doctors; and a trolley in the ED with equipment required for the Sepsis Six. The notes of all patients with lactate ≥2 mmol/l were retrospectively reviewed. Those with ≥2 systemic inflammatory response syndrome criteria and a documented suspicion of infection were deemed to have severe sepsis. The times at which these patients had each of the Sepsis Six completed were recorded, as were the final diagnosis and 7/28 day mortality.

## Results

In Cycle 1, 31/106 patients met the criteria for severe sepsis, compared with 36/120 in Cycle 2. The delivery of the Sepsis Six interventions was highly variable. In Cycle 1 lactate levels and i.v. access had the highest 60-minute completion rates (90.3%, 83.9% respectively). Blood cultures and i.v. fluid resuscitation were completed for 61.3% and 64.5% of patients within 60 minutes. Only 38.7% of septic patients were given i.v. antibiotics within 60 minutes. In total, 58.9% of patients received antibiotics in accordance with trust guidelines. High flow oxygen, catheters and fluid balance charts had the lowest 60-minute completion rates (35.5%, 6.5%, 6.5% respectively). In Cycle 2, post intervention, there was no significant change in the percentage of patients receiving the Sepsis Six bundle.

## Conclusion

The low rates of Sepsis Six completion require improvement to meet the targets set out by the College of Emergency Medicine. Our results suggest that simple interventions are ineffective in increasing Sepsis Six completion and thus lend support to the case for integrated interventions such as electronic recording and alert systems.
